# CNValidator: validating somatic copy-number inference

**DOI:** 10.1093/bioinformatics/bty1022

**Published:** 2018-12-12

**Authors:** Lucian P Smith, Jon A Yamato, Mary K Kuhner

**Affiliations:** Department of Genome Sciences, University of Washington, Seattle, WA, USA

## Abstract

**Motivation:**

CNValidator assesses the quality of somatic copy-number calls based on coherency of haplotypes across multiple samples from the same individual. It is applicable to any copy-number calling algorithm, which makes calls independently for each sample. This test is useful in assessing the accuracy of copy-number calls, as well as choosing among alternative copy-number algorithms or tuning parameter values.

**Results:**

On a dataset of somatic samples from individuals with Barrett’s Esophagus, CNValidator provided feedback on the correctness of sample ploidy calls and also detected data quality issues.

**Availability and implementation:**

CNValidator is available on GitHub at https://github.com/kuhnerlab/CNValidator.

**Supplementary information:**

Supplementary data are available at *Bioinformatics* online.

## 1 Introduction

Studies of somatic variation within organisms, particularly in neoplasia and cancers, often require inference of somatic changes in copy number. Such inference can be based on SNP array data using programs such as ASCAT (Van Loo *et al.*, 2010) or ABSOLUTE (Carter *et al.*, 2012), or on sequencing data using programs such as ascatNgs (Raine *et al.*, 2016). If copy-number inference is inaccurate, downstream conclusions may be incorrect.

We present the *haplotype coherency test*, which leverages information from multiple samples from the same patient to estimate the accuracy of inferred allele-specific copy-number calls. This test assumes that cross-sample haplotype coherency was not considered in the calls, which is true for most copy-number calling algorithms.

**Fig. 1.**
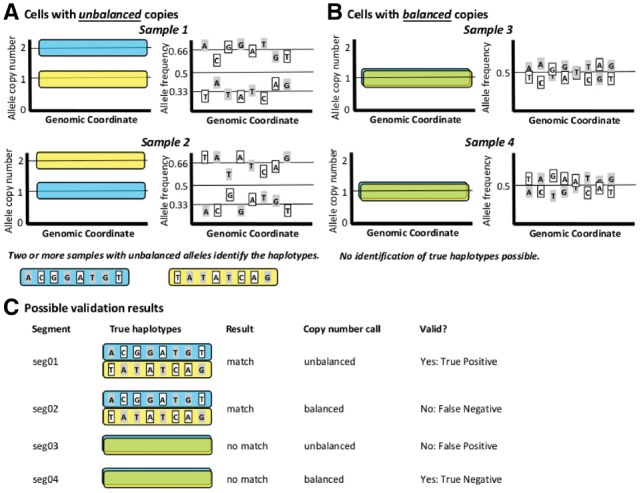
Haplotype coherency test. **(A)** Allele frequencies in unbalanced samples reveal the underlying germline haplotypes. **(B)** Balanced samples show only random fluctuation in allele frequencies and do not reveal the underlying haplotypes. **(C)** Once haplotypes are established, unbalanced samples should match the established allele frequency pattern, whereas balanced samples should show a randomized pattern

The test is illustrated in Figure 1. An individual’s germline is assumed to have two haplotypes (A and B) distinguished by the alleles present at heterozygous sites. We assume that as part of copy-number calling, the genome has been divided into segments of presumed constant copy number, and each segment has been assigned counts of the A and B haplotypes.

If a somatic event has generated a segment with more copies of one haplotype than the other (such as a single-copy gain or loss) we will term it ‘unbalanced’. An unbalanced region identifies which alleles are present on each haplotype: the allele frequencies of alleles on the more frequent haplotype will be shifted upwards, and those on the less frequent haplotype will be shifted downward. In contrast, in a ‘balanced’ segment (equal numbers of A and B haplotypes, such as a normal diploid), the pattern of allele frequencies will be due to noise and will not reflect the underlying haplotypes.

Thus, we expect that if a calling algorithm assigns an unbalanced call to a segment, the haplotypes indicated by the pattern of allele frequencies should match those in other samples where the segment is also unbalanced. If the haplotypes do not match, the call is likely wrong. Conversely, if a calling algorithm assigns a balanced call, the haplotypes should not match those in other samples. If the haplotypes do match, the call is likely wrong. Only the balanced/unbalanced status of the call is verified, not the exact call made: a miscall such as 2A/2B for 1A/1B cannot be detected.

The test is implemented by identifying heterozygous positions in the germline using a control sample. Only segments which span at least 10 germline heterozygous positions can be used. Each position is then scored as being above or below 0.5 in each somatic sample. For each segment, two samples are considered to agree on the underlying haplotypes when 95% or more of their heterozygous positions vary in the same direction—i.e. when an allele is high in one sample, it is high in the other, indicating that the same haplotype is preponderant in both samples. They also agree when 95% or more of the heterozygous positions vary in opposite directions, indicating that the A haplotype is preponderant in one sample and the B haplotype in the other. (For the rationale behind these cutoffs see Supplementary Methods.)

If no two samples agree on the haplotypes, no validation of the segment is possible. If 2+ samples agree, the true haplotypes are considered to be established, and the call made for each sample can now be evaluated. An unbalanced call which agrees with the established haplotypes is a true positive (TP), and one which disagrees is a false positive (FP). A balanced call which disagrees with the established haplotypes is a true negative (TN) and one which agrees is a false negative (TN). The *accuracy* of each sample’s calls overall is calculated as (TP+TN)/(TP+TN+FP+FN). This can be scored either by segment count or segment length: CNValidator implements both methods. Segments without established haplotypes or lacking 10+ germline heterozygous positions are annotated as unknown and omitted from calculation of accuracy.

CNValidator requires B-allele frequencies from array or sequencing data (though we have practical experience only with array data), and segments and copy-number calls from a copy-number algorithm. It uses simple input formats which can be easily prepared from a variety of file formats. It produces overall reports on the accuracy of each sample, and detailed reports covering each segment. It can be run either to validate a single copy-number algorithm, or to compare two or more algorithms or sets of algorithm parameters; in the latter case, it evaluates the union of segments called by all of the tested algorithms. It is written in Python 2 and is available under the MIT License.

## 2 Application

We developed this algorithm to validate calling on a mixture of Illumina 1.0 and 2.5 M SNP array data for 3–30 samples per individual from 210 individuals with Barrett’s Esophagus (BE) from the Seattle BE Study (Li *et al.*, 2014 and additional unpublished data). We performed segmentation with a customized version of copynumber (Nilsen *et al.*, 2012) and copy-number calling with a customized version of ASCAT (Martinez *et al.*, 2018; Van Loo *et al.*, 2010) (see Supplementary Material). For each sample, ASCAT was used to find the best ploidy estimate below 2.80 (‘low-ploidy’) and the best estimate 2.80 and above (‘high-ploidy’). Our goals were to validate copy-number calling overall and also to determine which ploidy baseline was preferable for each sample. We report only on the 1654 samples for which ASCAT found solutions for both baselines and at least one segment was validatable, and omit eight patients who either lacked solutions in either category, or had no validatable segments due to lack of copy-number variation.

For most samples (932/1654) both baselines had accuracy over 90%, reflecting the difficulty of distinguishing a cleanly genome-doubled sample from a diploid one (Supplementary Fig. S1). However, for 402 samples one solution was clearly preferable (260 low- and 142 high-ploidy). For example, sample 1005-24 100 had a low-ploidy accuracy of 33% and high-ploidy of 96%. Examination of individual calls showed many segments with inferred fractional copy number that would round to a balanced call with a low-ploidy baseline, but to an unbalanced call with a high-ploidy baseline; the coherency test strongly favored the unbalanced calls and thus the assignment of a high-ploidy baseline for this sample.

Accuracy was below 90% for both baselines for 320 samples. This may indicate high subclonality or noisy data. One striking case was patient 572. External evidence suggested high-ploidy solutions, but the inferred accuracy of these solutions was low (33–93%, see Supplementary Table S1). Quality control checks showed that this patient had been run using the wrong normal control; when the analysis was repeated with the correct control, accuracies were over 98%.

## 3 Discussion

Our BE results show the usefulness of CNValidator both in choosing among alternative copy-number approaches (in our case, low- versus high-ploidy baseline) and in detecting failure of copy-number calling, in our case due to a quality-control issue.

The approach used by CNValidator applies only to multiple samples from the same individual. In principle population-based haplotype inference could be used to validate single samples as is done by the Battenberg algorithm (Nik-Zainal *et al.*, 2012), although this would be vulnerable to errors in the inferred haplotypes. CNValidator requires at least two somatic samples but is more powerful with more samples (see Supplementary Fig. S3). Regions of subclonal copy-number variation can cause disagreement between the calling algorithm and CNValidator; CNValidator will sometimes detect unbalanced states from subclones that are not present in the majority clone. More work is required to assess the performance of CNValidator in the face of subclonality. Finally, CNValidator relies on the segmentation it is given; a separate approach will be needed to detect segmentation failures.

## Funding

This work was supported by National Institutes of Health [R01 CA61202-07 and P01 CA91955-11 to Brian Reid; R01 CA140657 to Carlo Maley].

## Supplementary Material

bty1022_Supplementary_Material
