# Supplementary material for: CNValidator: validating somatic copy-number inference
Source: Bioinformatics. 2018 Dec 12;35(15):2660–2. doi: 10.1093/bioinformatics/bty1022 (PMC6662281; doi:10.1093/bioinformatics/bty1022)
Supplement: bty1022_Supplementary_Material [file bty1022_supplementary_material.pdf]

Supplementary Material for "CNValidator: validating somatic copy-number inference"

Lucian P Smith, Jon A Yamato, Mary K Kuhner

Supplemental Methods

This section gives additional details of CNValidator's algorithm.

Selecting germline heterozygous positions. We defined "heterozygous" as $0.4 < \text{BAF} < 0.65$ for our Illumina SNP array data based on the shape of the BAF distribution in normal samples, but found that the exact cutoff made little difference as sites with borderline BAF were rare. A different set of cutoffs may be necessary if the data are based on WGS, especially if there is a bias towards calling of the reference allele.

Regions of known germline copy number variation should be excluded if possible, as the test assumes that germline contains exactly two distinct haplotypes. However, we did not attempt this for our practical example as our pipeline did not call such variants.

A small proportion of germline homozygous sites will have been typed as heterozygous in the normal control due to experimental error or somatic mutations in the control tissue. These will tend to be homozygous for the same allele in all tumor samples (because they were homozygous in the ancestral germline) and will generate sporadic spurious matches. We handle this by requiring that a segment to be validated must have 10 or more heterozygous sites, and defining matching as 95% agreement of the inferred haplotypes. Sporadic false matches will generally not reach this threshold.

The cutoff of 10 sites was chosen because a false match across 10 sites with random data is expected to occur with a frequency of 1/512, and false matches are therefore expected less than once per sample for the segmentation we used. This value can be changed as a program constant in CNValidator (see documentation). The cutoff at 95% was chosen empirically to give good separation between correct and incorrect segments; it can also be changed as a program constant. We found technology-dependent excesses of segments with 60-85% matching in our array data (data not shown); the 95% cutoff avoids false matching generated by this effect. It is possible that a genuine unbalanced segment in a noisy sample could be marked as a false positive because its putative haplotypes are not similar enough to those in other, less noisy samples, but adjusting the cutoffs to reduce the chance of this necessarily increases the chance of calling a true positive due to accidental matching.

In Illumina 1.0M SNP array data, some positions were included on the array to probe areas of population copy-number variation and are expected to be homozygous (these have probe names beginning with "cnvi"). As these positions were not designed to provide BAF data, we excluded them from use in valida-

tion. Other data platforms may have similar categories of sites which should be omitted.

Defining increased or decreased B-allele frequency (BAF) in the somatic sample. We initially assumed that it would be best to score only positions which were very far from 0.5 in the somatic sample, but found that doing so actually amplified the effect of errors (such as sites that were actually homozygous in the germline) as such errors tend to produce extreme BAFs. The result of this amplified error was a high rate of unexpected haplotype agreement for regions where one or both samples were called as balanced. More robust results were produced when we scored all BAFs above 0.5 as increased and all BAFs below 0.5 as decreased. This preserved matching in unbalanced/unbalanced cases while greatly reducing probably-spurious matching in cases where one or both calls were balanced.

If the modal BAF at heterozygous sites is not 0.5, it may be necessary to change this definition. An example would be sequencing data with a bias towards the reference allele. The mode of the BAF distribution would be a reasonable choice for the increase/decrease breakpoint, or sites could be scored in the context of whether the increased allele was reference or non-reference. As this will vary with every technology, we have not implemented such a correction in CNValidator.

Reconciling segment boundaries. The haplotype coherency test assumes that the segment boundaries are the same across all samples and calling algorithms. This will automatically be the case if a joint segmentation algorithm such as the *multipcf* routine of *copynumber* (Nilsen *et al.*, 2012, 2013) or the joint segmentation algorithm of *ASCAT* (Van Loo *et al.*, 2010) version 2.4+ is used. It is not valid to compare accuracy scores across two different segmentations. To see why, consider a set of samples with modestly sized unbalanced regions. If the segmentation divides these regions from the surrounding balanced material, we can assess whether a caller correctly identifies them as unbalanced. If the segmentation lumps them with the remainder of the chromosome and the caller identifes the whole chromosome as balanced, validation will not detect this problem: the short imbalanced regions will not be enough to trigger validation failure as they will be swamped by the remainder of the chromosome. Therefore, comparisons should be of different calling strategies across a common segmentation.

This could be dealt by superimposing a single set of segments on the output from both algorithms: either the segments produced by one of the algorithms, or a fine-grained default segmentation. We used this approach when testing different values of the gamma parameter in *copynumber:* segmentation with one specific value of gamma was used as the basis for all comparisons.

For the CNValidator program we did not want to assume availability of a good "default" segmentation. Instead, we used the union of all segmentation breakpoints from all algorithms under comparison. This can create many short segments which cannot be validated, especially if a large number of algorithms are being simultaneously compared, but in practice we have had acceptable performance for comparing 2-3 algorithms. If users wish to evaluate multiple algo-

rithms on a segmentation derived from just one of them, as we did for gamma values, this can be done with CNValidator by pre-processing the segmentation data to superimpose a common set of segments.

An algorithm somewhat similar to CNValidator has been used by Xia *et al.* (2014) to detect subclonality in somatic samples.

Data Generation and Analysis

Biological data set. To explore the usefulness of the test, we used data collected from individuals with Barrett's Esophagus (BE) by the Seattle Barrett's Esophagus Study. The Seattle Barretts Esophagus Study has been approved by the Fred Hutchinson Cancer Research Center Institutional Review Board IRB# 8344. Patients enrolled in the study signed research consent. Epithelial isolated samples were run on Illumina 1.0M or 2.5M arrays for copy number inference. The 1.0M array data comes from the study described in Li *et al.* (2014); the 2.5M array data comes from a study in preparation (Brian Reid, personal communication).

We performed segmentation using a version of the *copynumber* library (Nilsen *et al.*, 2012, 2013) with two modifications. (1) We modified the *multipcf* routine to handle missing data without imputation. This allowed us to combine data from different array platforms. (2) *Copynumber* uses a gamma parameter to determine how aggressively to break up the sequence. Natively, *copynumber* rescales gamma based on the number of samples, but in order to fairly compare results from individuals with different numbers of samples, we disabled this rescaling. All gamma values mentioned in this paper are non-rescaled values. Our modified version of *copynumber* is available at `https://github.com/kuhnerlab/copynumber`

To accommodate data from multiple SNP array types, we used the following rules. All SNPs with the same name and position in both arrays were used. When the two arrays disagreed on the position of a SNP, the metadata from the 2.5M array were used as these are more recent and presumably more accurate. SNPs coded as having an unknown location in the 2.5M array metadata were dropped from analysis. When a SNP appeared on one array but not the other, it was coded as missing data in samples run on the other array. Missing data were not imputed.

We performed segmentation using the modified *copynumber* jointly over all array samples for each patient. We determined a suitable value of the gamma parameter via a preliminary analysis where we tested gamma values from 100 to 3000, evaluating accuracy by superimposing all copy number calls onto the segmentation produced by gamma = 100 (data not shown) and measuring per-megabase accuracy. This approach recommended gamma = 500, which we used in all futher analyses.

We assigned allele-specific copy numbers using code derived from the modified version of *ASCAT* described in Martinez *et al.* (2018). We additionally modified this version of *ASCAT* to allow ploidy to be constrained in a specific range,

so that we could obtain multiple solutions. *ASCAT's* plots of goodness of fit as a function of purity and ploidy generally indicated two high-performing solutions, one close to diploid ("low-ploidy") and one close to tetraploid ("high-ploidy"); additionally, flow-cytometric data shows that near-diploid and near-tetraploid clones predominate in BE (Rabinovitch *et al.*, 2001). We constrained values of ploidy in ASCAT by constraining its internal constant "psi" (essentially ploidy plus a rescaling factor). Internally, ASCAT performs a grid search of psi values, moving in increments of 0.05, and rejecting maxima found in the three "edge-most" values of the search. We constrained psi for our low-ploidy search to a grid of values from 1.0 to 2.9, meaning that maxima were constrained between 1.15 and 2.75 (inclusive). For a high-ploidy constrained search, we restricted psi to values from 2.65 to 6.0, meaning that maxima were constrained between 2.80 and 5.85. These constraints prevented ever obtaining the same maximum for both sources. Note that both solutions used the same underlying segmentation, therefore could be directly compared. In some cases, only one of the two searches found a maximum; we do not report further on these cases. Our modified version of *ASCAT*, based on previous modifications by Pierre Martinez (Martinez *et al.*, 2018) is available at `https://github.com/kuhnerlab/p_ascat`.

Supplementary Results

In Figure S1 we show the haplotype coherency accuracy score for low-ploidy and high-ploidy solutions for all 1654 samples in which both solutions were produced. (In the remaining cases, either our modified *ASCAT* failed to generate a solution for one of the constraints, reflecting a search space with no maximum inside the constraint area, or no segments could be validated due to lack of copy-number variation.)

For the majority of samples accuracy was high and similar for low-ploidy and high-ploidy baseline solutions, reflecting the known difficulty of distinguishing a cleanly genome doubled sample from a diploid one with this technology. However, CNValidator showed accuracy above 90% for one baseline and below 90% for the other in 402 samples, which we interpret as guidance on which baseline is likely correct. It also indicated a likely failure of copy-number calling (accuracy below 90% for both baselines) in 320 samples. Manual examination of the array data was generally concordant with the CNValidator results (data not shown).

Figure S2 addresses the question of whether accuracy is correlated with estimated purity (the proportion of the sample estimated by our modified ASCAT to consist of BE cells). No relationship between purity and accuracy was seen.

The next three figures consider whether genomic coverage (the proportion of the genome which could potentially be validated, which requires segments with sufficient heterozygous SNPs, and matching of the SNP pattern between at least two samples) correlates with other measures of the amount of information present for an individual patient.

Figure S3 addresses the relationship between the number of samples and mean coverage. Additional samples should improve the proportion of the genome

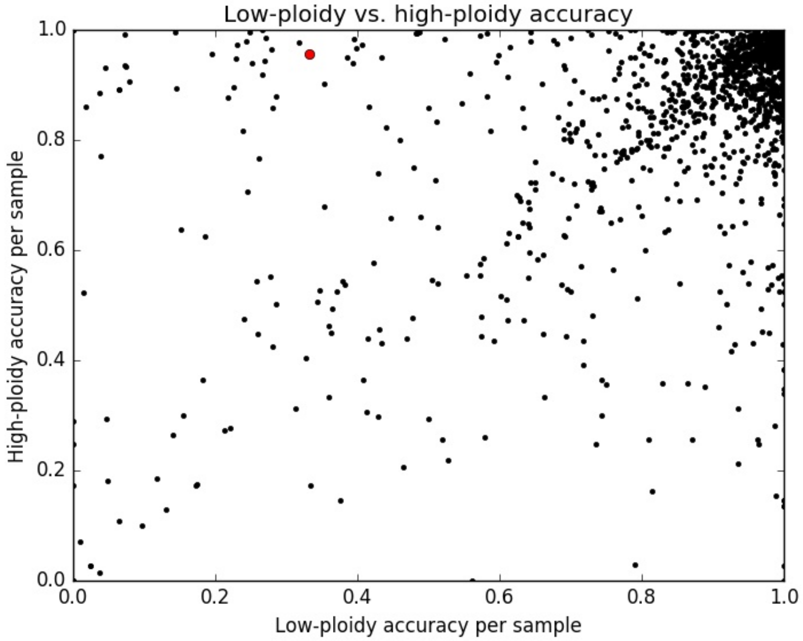

Figure S1: Per-megabase accuracy scores for low-ploidy and high-ploidy baselined calls. Red dot shows sample 1005-24100, discussed in main text, for which validation clearly prefers the high-ploidy solution.

that can be validated, as there is more chance to detect overlapping copy-number variants in two or more samples; a weak relationship in the expected direction is seen. For most of our BE samples, only a small proportion of the genome could be validated due to limited copy-number variation.

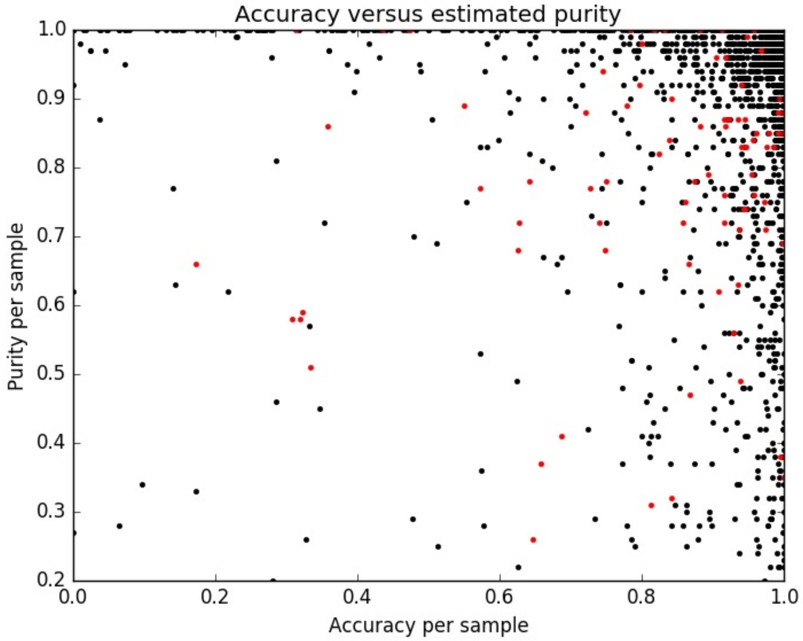

Figure S2: Per-sample accuracy score versus sample purity as estimated by ASCAT. Accuracy is computed per megabase. Black samples were inferred to be low-ploidy (estimated ploidy <= 2.75) and red samples were inferred to be high-ploidy (estimated ploidy >= 2.8). Regression was non-significant (p=0.739) and is not shown.

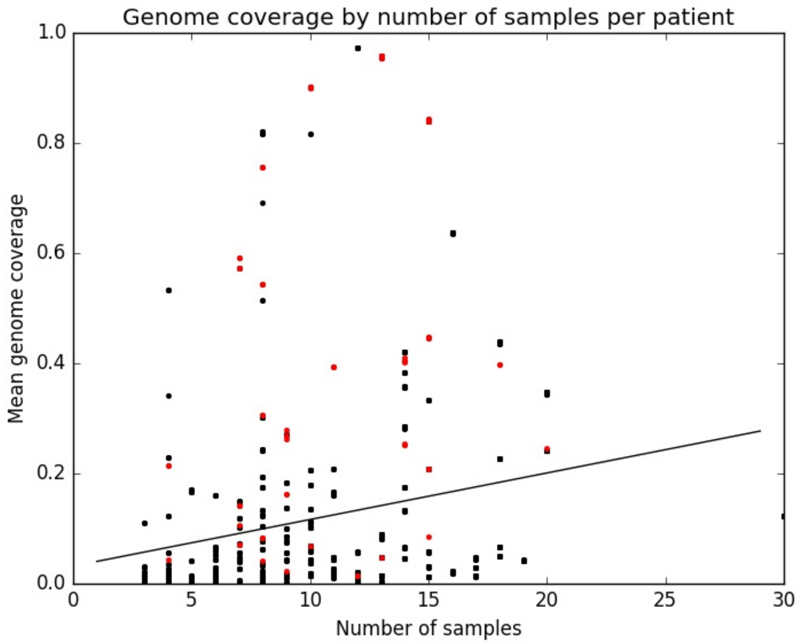

Figure S3: Number of samples for a given patient versus the percentage of the genome that could potentially be validated in samples from that patient. Black dots indicate patients for whom all samples were inferred to be low-ploidy; red dots indicate patients for whom one or more samples were inferred to be high-ploidy. Black line shows regression for all data, $R^2 = 0.047$, $p < 0.0001$.

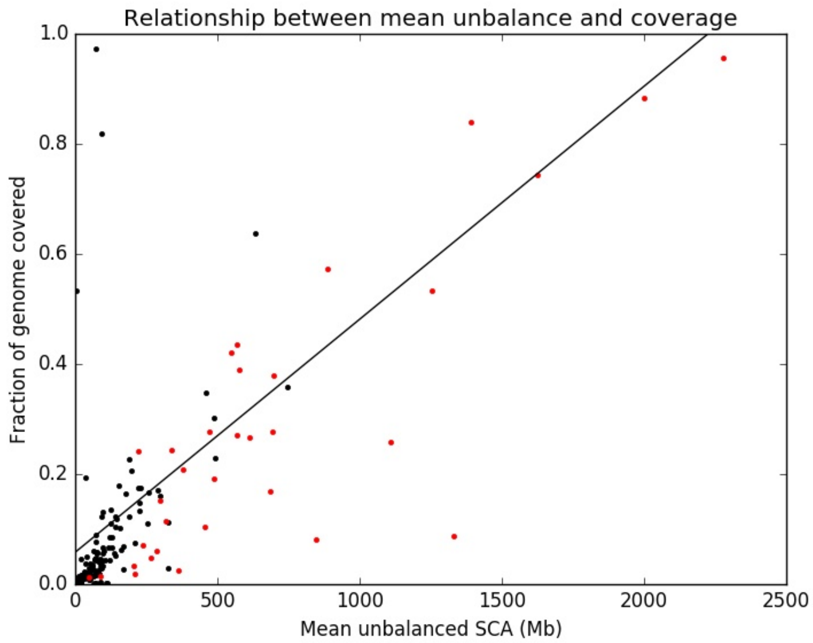

Figure S4: Mean Mb inferred as unbalanced, averaged across samples from a patient, versus proportion of the genome that could potentially be validated in samples from that patient. Black dots indicate patients for whom all samples were inferred to be low-ploidy; red dots indicate patients for whom one or more samples were inferred to be high-ploidy. Black line shows regression for all data, $R^2 = 0.590$, $p < 0.0001$.

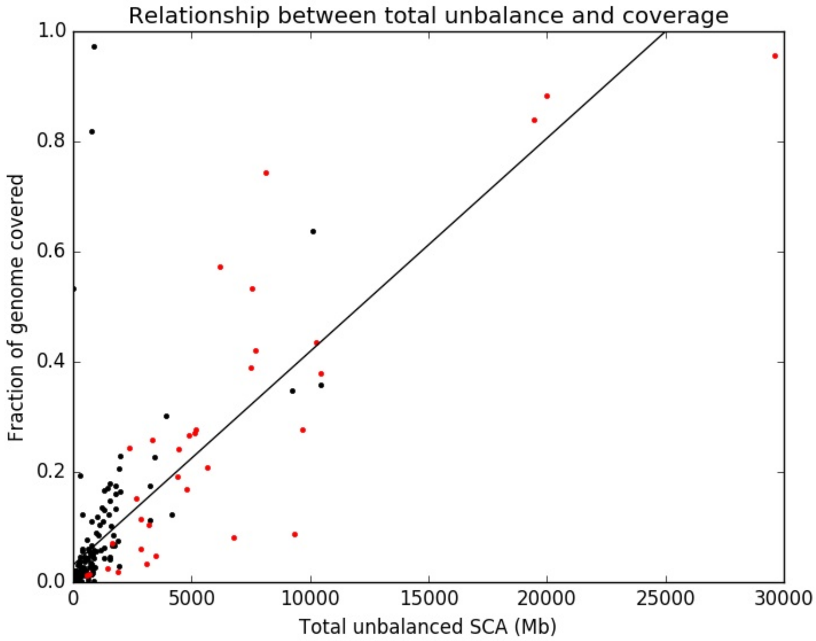

Figure S5: Mean Mb inferred as unbalanced, totalled across samples from a patient, versus proportion of the genome that could potentially be validated in samples from that patient. Black dots indicate patients for whom all samples were inferred to be low-ploidy; red dots indicate patients for whom one or more samples were inferred to be high-ploidy. Black line shows regression for all data, $R^2 = 0.590$, $p < 0.0001$.

Table S1: Inferred accuracy of high-ploidy solutions for patient 572 with incorrect and correct normal control used in copy-number calling.

Sample	High-ploidy accuracy	
	Incorrect normal	Correct normal
23785	0.814	0.997
23779	0.812	0.997
24741	0.330	0.996
23776	0.770	0.997
21368	0.468	0.990
21370	0.933	0.978
21362	0.477	0.987
21364	0.482	0.987

Figures S4 and S5 address the relationship between the proportion of the genome that was called as unbalanced by ASCAT and the proportion of the genome that could potentially be validated. A positive relationship would be expected, as unbalanced segments are more likely to generate the matches needed to make a segment potentially validatable. Figure S4 considers the mean length of unbalanced calls per genome averaged across samples from a patient. Figure S5 considers the total length of unbalanced calls, accumulated across samples from a patient; this measure depends on the number of samples as well as the amount of unbalanced material in each sample. In both cases, the expected increase in proportion of the genome that can be validated as the proportion of the genome called as unbalanced increases is seen, though the relationship is noisy. Samples called as high-ploidy had larger proportions of the genome which could potentially be validated than those called as low-ploidy.

During the development of CNValidator, we noticed poor validation for samples from patient 572 run with a high-ploidy baseline, despite external evidence that most of these samples should be high-ploidy. Investigation showed that copy number inference had used a normal control from a different patient. For this paper, we re-ran the samples from patient 572, high-ploidy baseline, with the incorrect and correct normal controls using our final version code and pipeline. Results are shown in Table S1. CNValidator successfully detected a quality control issue for this patient.

We end by describing an issue with ASCAT that was uncovered by CNValidator. In some segments, ASCAT inferred non-integer copy numbers that were identical to each other, but summed to an odd number. It always returned unbalanced calls in such cases; for example, if A and B were both estimated with copy number 1.5, a call of 1A/2B or the reverse was returned, with an arbitrary choice of which haplotype was increased. Using CNValidator, we found that many of these calls should have been balanced, and corrected them accordingly.

LITERATURE CITED

References

Li,X. *et al* (2014) Temporal and spatial evolution of somatic chromosomal alterations: a case-cohort study of Barrett's esophagus, *Cancer Prev. Res.*, **1**, 114-127.

Martinez,P. *et al* (2018) Evolution of Barrett's espohagus through space and time at single-crypt and whole-biopsy levels, *Nat. Comm.*, **9**, article 794.

Nilsen,G. *et al* (2012) Copynumber: Effecient algorithms for single- and multi-track copy number segmentation, *BMC Genomics*, **13**, 591.

Nilsen,G. *et al* (2013). copynumber: Segmentation of single- and multi-track copy number data by penalized least squares regression. R package version 1.20.0.

Rabinovitch,P.S. *et al* (2001) Predictors of progression in Barrett's Esophagus III: baseline flow cytometric variables. *Am J Gastroenterol*, **96**, 3071-3083.

Van Loo,P. *et al* (2010) Allele-specific copy number analysis of tumors, *Proceedings of the National Academy of Sciences*, **107**, 16910-16915.

Xia,R. *et al* (2014) Identification of allelic imbalance with a statistical model for subtle genomic mosaicism. *PLoS Comput Biol*, **10**, e1003765.

Documentation

User Documentation: CNValidator

Jon Yamato and Mary Kuhner
June 21, 2018

Overview

This program assesses the likely validity of somatic copy number calls, based on two or more somatic samples from the same patient and a normal control sample. The somatic samples need not be cancer; they could be neoplastic or benign or even healthy tissues. However, the test will be uninformative unless there is shared somatic copy-number variation among at least some of the samples. For example, it is unlikely to be informative if just one sample is taken from each of several unrelated tumors. It uses the *haplotype coherency test* as described in:

Smith LP, Yamato JA, Kuhner MK (2018). CNValidator: validating somatic copy-number inference. Bioinformatics submitted.

The program, documentation, and test files can be found at:

https://github.com/kuhnerlab/CNValidator

Briefly, the logic of the test is that if the pattern of germ-line heterozygous sites with increased or decreased allele frequencies corresponds between two or more samples for the same genomic segment, that segment should have been given an unbalanced copy-number call for those samples; and samples which do not show correspond to this shared pattern should have been given a balanced copy-number call.

CNValidator checks the internal consistency of a pipeline that first divides the genome into segments believed to have the same copy number ("segmentation") and then assigns integer allele-specific copy number calls for each segment. It assumes that cross-sample haplotype information was not used to inform the copy-number calls. (If haplotype coherency was considered in making the calls, considering it when validating the calls will not add anything; probably all calls will be judged valid.)

It is written in Python 2 and has been tested on version 2.6. Minor changes to print statements should allow it to run in version 3 if desired.

It can be run in two ways:

(1) If only one copy number calling algorithm is used, it will assess the proportion of copy-number calls which passed the coherency test.

(2) If more than one copy number calling algorithm is used, a new set of segments will be generated as the union of segment breakpoints across all algorithms, and the proportion of these segments which are valid based on the calls from each of the algorithms will be assessed. This allows multiple algorithms to be assessed on the same underlying segmentation.

In both cases, some proportion of calls will not be validatable; the proportion of validatable calls depends on the structure of the data, especially the number of samples, but also the frequency of somatic copy-number variants and the degree to which they are shared across samples.

Approach (1) is useful in determining whether specific calls, or the copy-number calling as a whole, are likely to be correct. Approach (2) is useful in deciding among multiple competing copy number calling algorithms, parameter choices, or other variable factors. For example, it can be used to determine whether treating a sample as low-ploidy or high-ploidy yields superior copy number calls.

We define five outcomes for examinination of a segment in a given sample:

Unvalidatable (UN). The segment cannot be validated. This can be due to too few samples having copy-number variation across the segment; not enough heterozygous positions in the normal sample; or too much missing data. No conclusion can be drawn about whether the copy number calls for this segment are correct.

True Positive (TP). The segment was called as unbalanced (unequal numbers of the two haplotypes) and the coherency test agrees that it is unbalanced.

False Positive (FP). The segment was called as unbalanced, but the coherency test suggests that it is balanced.

True Negative (TN). The segment was called as balanced (equal numbers of the two haplotypes) and the coherency test agrees that it is balanced.

False Negative (FN). The segment was called as balanced, but the coherency test suggests that it is unbalanced. Data that show a high proportion of FN results can arise from the presence of unbalanced events that are subclonal (present in only a fraction of the cells in the sample). Subclonal events which fall below the detection threshold of the copy number calling algorithm may still produce enough signal to be seen as unbalanced by the haplotype coherency test.

These outcomes are combined into an accuracy score:

A = TP + TN / (TP + TN + FP + FN)

The program assesses accuracy on both a per-segment and per-megabase basis. The per-segment accuracy indicates what proportion of segments are likely to be called correctly; the per-megabase accuracy indicates what proportion of the genome is likely to be called correctly. These can be quite different if, for example, calls of long segments are reliable while calls of shorter segments are noisy.

The higher the accuracy score, the better the quality of the copy number calling (though the score should be interpreted cautiously if very few segments could be validated). For human 2.5M Illumina SNP array data we found that accuracies below 90% suggested a problem with data quality or an incorrect ploidy call. When comparing two different calling algorithms or settings (such as solutions assuming low versus high ploidy), the higher-accuracy solution is preferable, but no significance test is available to determine whether the accuracy difference is statistically significant.

The program also reports the outcome (UN, TP, TN, FP, FN) for each segment in each sample. This can assist the user in judging which calls may be

unreliable, especially in genomic regions of special interest.

Input

This program was tested on segmentation and copy number calls from SNP array data, but should be equally applicable to calls from DNA sequencing data. It operates on files from a single patient. The patient's germline is presumed to be diploid, though the somatic samples need not be. Regions in which the germline is not diploid should, if possible, be removed from the analysis; this includes Y chromosomes and X chromosomes of males.

The program has a command line interface with the only argument being the name of the master file. If the master file is not in the same directory or folder as the program, then a full path to the master file must be provided.

Four types of input files are required:

(1) A *master file* that enumerates and groups all of the other input files. The path to this master file is given as a command line argument to the program.

(2) A *normal-control BAF file* giving the B allele frequency (BAF) data for the sample representing the patient's germline. BAF is defined in this context as B/(A+B) and can be taken from SNP array output or allele-specific sequencing read counts.

(3) One or more *sample BAF files* giving BAF data for the somatic samples. Each sample may have its own sample BAF file or multiple samples may be present as separate columns in the same file.

(4) Multiple *segmentation files*, one per sample per copy-number algorithm to be tested. These files represent the output of a segmentation and copy number calling program such as ASCAT.

Sample input files for a small fictional data set are provided with the program.

Input File Formats

As there is no clear consensus on the format of BAF files or, especially, segmentation files, we have adopted a simple format which should be fairly easy to reach from a variety of common bioinformatics formats.

(1) The master file must contain blocks of entries corresponding to file types (2)-(4) above. The order of the blocks does not matter.

The block for the normal-control BAF file is as follows:

```
#NORMAL_BAF
path/to/baffile
```

The block for the sample BAF file or files is as follows:

```
#SAMPLE_BAF
path/to/firstbaffile
path/to/secondbaffile
```

```
...
```

There may be one or more than one block corresponding to segmentation files, depending on whether one or more than one copy-number algorithm is being tested. Each algorithm needs a separate segmentation block for which the second header line is a user-defined name for the algorithm. (This will be used in the output to identify the algorithm; short distinctive names work best.) For example, the following block presents segmentation files for the low-ploidy version of the copy-number algorithm:

```
#SEGMENTATION
#diploid
path/to/sample1/segmentation
path/to/sample2/segmentation
path/to/sample3/segmentation
...
```

(2) The normal BAF file represents BAFs for the normal (blood or healthy tissue) control for this patient. Such a sample is required as the haplotype coherency test is limited to using positions at which the patient's germ line was heterozygous, and this is determined based on the normal sample.

This is a tab-separated file of the following format:

The first line should be a header as follows (note that this line *starts with a tab* to meet expectations of R programmers):

```
    Chr Position [SampleName]
```

where [SampleName] represents a name for the normal sample. Subsequent lines should each contain BAF information for one position. Any positions not listed in this file will not be used in the coherency test even if they are present in the somatic samples.

The first entry (with no column header) is the name of the SNP at this position, or a "." if names are unavailable (as for WGS). In some Illumina SNP chip data, sites whose names begin with "cnvi" are present: these represent normally-homozygous sites in regions of population variation in copy number. Our program filters out such sites as they appear inappropriate for this test since they do not have B alleles in the conventional sense. SNP names are not required; they are maintained in our input format mainly for use in data quality checks.

The second entry is the chromosome number or name. Any convention for chromosome names may be used as long as it is consistent through all input files (for example, the human first chromosome could be called "1" or "chr1" as long as all files are consistent).

The haplotype coherency test should not be applied to data from haploid regions of the genome such as the Y, the X in a male, or mtDNA. As the program does not have the information to determine which genomic regions should be

removed, the user is responsible for removing them from the normal BAF file prior to running CNValidator.

The third entry is the chromosomal position as an integer. Any coordinate system may be used as long as it is consistent throughout all input files. Watch out for the difference between zero-based and one- based coordinates! In particular, BAM files are zero-based and VCF files are one-based; if the data originate from a mix of file types a conversion will be needed. CNValidator does not attempt to do this conversion as it has no way to know the origins of its data files.

The fourth and final entry is the BAF score for that position as a floating-point number between 0.0 and 1.0 inclusive. This could be the BAF estimate from SNP chip processing software, or the fraction of reads showing the B allele from sequencing data. It does not matter which allele is defined as B but for any given position the same allele *must* be defined as B in all samples, or the program's results will be meaningless. The BAF score may also be NA to indicate missing data; such positions will not be used for this sample's scoring, but may still be used for other samples for which data are available.

(3) The sample BAF file or files contain BAF values, defined as above, for the somatic tissue samples. One or more samples may be present in each somatic BAF file, but each sample must be in just one file, not split across multiple files.

The format for these files is the same as for the normal BAF file, except that it may have more than one column for the BAF values, each headed by the name of the sample to which those BAFs apply. For example, a file with two samples might start like this:

```
      Chr  Position  Sample1  Sample2
rs32  1    100       0.43     0.98
```

This represents a (fictional) position named rs32 on chromosome 1, position 100, which has a BAF of 0.43 in Sample1 and a BAF of 0.98 in Sample2.

As noted above, be careful of differences in coordinate systems, and make sure that the same allele is defined as the B allele across all samples. "NA" for a sample BAF value will cause that position to be disregarded for that sample, but it may still be used for other samples.

(4) The segmentation files must be one file per sample per algorithm. It is an error if any sample/algorithm combination is missing, or if the set of samples differs between the sample BAF files and the segmentation files.

These are tab-separated files with the following format:

The first line is a header as follows:

```
patient sample chr startpos endpos intA intB
```

The first column is a patient identifier, and must be the same in every segmentation file (as an error check against inclusion of material for a different patient, which spoils the test). The second column is the sample name, and must exactly match the corresponding sample name in the header of one of the sample BAF files. The third position is chromosome identifier, as before.

The fourth and fifth columns are the starting and ending genomic positions of the segment. These positions are understood to be inclusive on both ends (a closed interval): that is, both the position at *startpos* and the one at *endpos* are understood to be part of the segment. These positions do not have to be the positions of SNPs, nor to be mentioned in the BAF files.

The sixth and seventh columns are the allele-specific integer copy number estimates for this segment, with *intA* representing the A-allele copy number and *intB* representing the B-allele copy number. The designation of A and B is arbitrary. Both *intA* and *intB* must be greater than or equal to zero, or NA indicating missing data. (Segments with NA for either or both of intA and intB will be treated as unvalidatable for that sample.)

Output

The CNValidator program writes two types of output files.

(1) A single *Summary output file* is produced covering all results. The name of this file is "PID_overall_output.tsv" where PID is the user-specified patient identifier.

This is a tab-separated file with the following entries:

Sample: the user-specified sample name.

Method: the user-specified copy-number algorithm name (important when more than one algorithm is being compared in a run).

MB_total: the total genome length in the segments considered by the program, in megabases (Mb).

MB_validated: the total length of segments that were deemed valid.

MB_contradicted: the total length of segments that were deemed invalid.

MB_accuracy: the length-based accuracy score (MB validated/ MB validatable).

Segments_total: the total number of segments considered by the program.

Segments_validated: the number of segments that were deemed valid.

Segments_contradicted: the number of segments that were deemed invalid.

Segments_accuracy: the segment-based accuracy score (segments validated/ segments validatable).

Caution should be used in evaluating the accuracy scores if the length of genome and/or number of segments that could be validated is very low. For example, 100% validation is not impressive if only one segment could be validated. If no segments could be validated, the accuracy scores will be given as "NA".

(2) Multiple *detailed output files*, one per sample. These files show the score (TP, TN, FP, FN, UN) for each segment, for each copy-number algorithm.

The names of these files are "PID_SID_detailed_output.tsv" where PID is the patient ID and SID is the sample ID.

These are tab-separated files with the following entries:

chrom: chromosome of segment.

startpos, endpos: boundaries of segment. The segment is closed (contains both startpos and endpos). Note that when multiple copy-number algorithms with different segmentation are used, new segments will be produced representing the union of existing segment breakpoints. This may cause segments to start or end on positions which are not SNPs. For example, if one method saw a segment from 1-10, and a different method saw a segment from 1-20, this will result in output describing segments from 1-10 and 11-20, even if site 11 is not mentioned anywhere in the input data.

nBAFs: the number of sites heterozygous in blood that fall within this segment. This gives some information on how well-founded the finding of validation or non-validation is: ten BAF scores will occasionally vary in the expected direction by chance, whereas ten thousand BAF scores should not. Note that this number does not take into account missing data in the somatic samples, which may contribute to a segment being unvalidatable in a particular sample even though it could be validated in other samples.

These fields are followed by a pair of fields for each method being tested. The first field is called methodname_call (where methodname is the user-specified name of the calling method) and gives that method's allele specific copy number call, written as A/B. For example a call of 2/3 means that two A haplotypes and three B haplotypes were inferred to be present in this segment by this method.

The second field is called methodname_evaluation, and gives the validation result corresponding to that method. For example, if method 1 called a segment unbalanced, the evaluation will be either TP, FP, or UN, corresponding to the copy number call being valid, invalid, or unvalidatable.

Sample output files generated for the example input data are supplied with the program. As there is no stochastic component to this program, running the sample input files should exactly replicate the sample output files.

Program Parameters

At the top of the python program file (CNValidator.py) there are several program paramenters. The default values were chosen based on data derived from Illumina 1.0M and 2.5M SNP arrays, and if your data were derived in some other way, adjusting these parameters may be helpful.

The two parameters *min_useable_baf* and *max_useable_baf* give the limits within which a position will be considered to be heterozygous in the normal sample. Good values for these parameters can be found by making a histogram of BAF values; the *min_useable_baf* and *max_usable_baf* should fall in the valleys between heterozygous positions around 0.5 and homozygous positions around 0 and 1.

The list variable *badbafvals* contains the strings which will be considered to represent missing data (normally "NA"). If your data use a different value such as "?" for missing data, you can add it to this list.

The integer variable *min_bafcount* gives the minimum number of valid BAF positions within a segment in order for that segment to be validatable. A

valid BAF position must be heterozygous in the normal sample (as defined by *min_useable_baf* and *max_useable_baf* above) and non-missing in the somatic sample. A segment which has fewer than this number of valid BAF positions will be treated as unvalidatable in this sample. We do not recommend setting this value below its default of 10, but values above 10, which lead to treating a larger proportion of short segments as unvalidatable, might be useful for very noisy data such as DNA data from a degraded sample.

The variable *min_matching* defines the degree of agreement between the BAF pattern in two samples required to consider them as representing the same haplotypes. For example, the default value of 0.95 means that 95% of the BAF values (at sites heterozygous in normal) must co-vary in the same direction, or 95% must co-vary in the opposite direction, in order for the BAF patterns to be considered as indicating the same haplotypes. This value must be strictly greater than 0.5 for the test to work at all. If the user wishes to be particularly skeptical of unbalanced calls, *min_matching* can be set higher, but this will render the test less sensitive to unbalanced regions that were called as balanced. If the user wishes to spot as many missed unbalanced regions as possible, or for very noisy data, *min_matching* can be set lower, but we recommend setting *min_bafcount* higher if this is done, to avoid spurious matching in short segments. For example, if *min_matching* were reduced to 0.90, *min_bafcount* should be increased, perhaps to 20.

The list variable invalid_chromosomes lists the names of chromosomes which should not be used in validation, most commonly because they are not diploid (e.g. Y, X in a male, mtDNA). Any chromosome names inserted into this list will be disregarded; conversely, the X chromosome can be removed from this list in order to allow validation of X chromosome segments, though this is only appropriate if all samples included in validation come from females. If both males and females are present in the file, the X chromosome should be excluded, but this will prevent validation of female X chromosome data; if such validation is desired, a separate run with only females can be performed, or males can be coded as missing data for X chromosome segmentation.

Authorship and Contact Information

This program was written by Jon Yamato and Mary Kuhner based on code by Lucian P. Smith. It is released under the MIT License (see the LICENSE file distributed with the software). We would be delighted to hear about any projects or publications using this code.

The authors can be contacted with questions, bug reports, or suggestions for improvement at:

Mary Kuhner
Department of Genome Sciences, University of Washington
Box 355065
Seattle, WA 98195-5065
mkkuhner@uw.edu
